# Real-World Study of Adding Bevacizumab to Chemotherapy for Ovarian, Tubal, and Peritoneal Cancer as Front-Line or Relapse Therapy (ROBOT): 8-Year Experience

**DOI:** 10.3389/fonc.2020.01095

**Published:** 2020-07-14

**Authors:** Pei-Ying Wu, Ya-Min Cheng, Meng-Ru Shen, Yi-Chun Chen, Yu-Fang Huang, Cheng-Yang Chou

**Affiliations:** ^1^Department of Obstetrics and Gynecology, National Cheng Kung University Hospital, College of Medicine, National Cheng Kung University, Tainan, Taiwan; ^2^Department of Medicine, College of Medicine, National Cheng Kung University, Tainan, Taiwan; ^3^Department of Pharmacology, College of Medicine, National Cheng Kung University, Tainan, Taiwan

**Keywords:** ovarian cancer, fallopian cancer, peritoneal cancer, bevacizumab, progression, survival, drug-related side effects, platinum sensitivity

## Abstract

This study aimed to determine the real-world, long-term prognostic impacts, and adverse effects (AEs) of bevacizumab (BEV) in Asian patients with ovarian/tubal/peritoneal cancers. We retrospectively reviewed the medical records of consecutive patients with ovarian/tubal/peritoneal cancer on front-line chemotherapy with or without BEV (Cohort 1) and those who relapsed following chemotherapy and/or BEV (Cohort 2) between 2011 and 2018 in a tertiary medical centre. Patient characteristics, BEV dosages, clinical outcomes, and AEs were analyzed. Hazard ratios for disease progression and death were analyzed using a cox proportional regression model. Benefits of BEV used throughout triweekly, in terms of improved progression-free survival (PFS) and overall survival (OS), were observed at a dosage of 7.5–15 mg/kg among advanced-stage Cohort 1 patients. A progression-free interval of <6 months was the strongest predictor of disease progression and death in advanced-stage patients. BEV throughout and optimal cytoreduction were independent predictors of reduced disease progression. No prognostic advantage was observed between serous and clear cell histologies when BEV was added. Moreover, BEV resulted in improved OS in Cohort 2 patients, especially in the platinum-sensitive subgroup. Most patients had a front-line BEV dosage <10 mg/kg per cycle with <10 treatment cycles. Low rates and grades of BEV-related AEs were observed in both cohorts. BEV used throughout effectively extended PFS and OS in advanced-stage patients with ovarian/tubal/peritoneal cancer. Patients with platinum-sensitive carcinoma, treated with BEV, had a significant improvement in OS and extended PFS. Therefore, BEV can safely be added to chemotherapy for ovarian/tubal/peritoneal cancers.

## Introduction

Epithelial ovarian cancer (EOC) is the leading cause of death among gynecological cancers in many countries (1–3). Most patients with chemotherapy-sensitive disease develop chemotherapy-resistant relapse after undergoing several lines of therapy. Maintenance therapy with targeted agents following primary cytoreductive surgery and chemotherapy to extend the progression-free interval (PFI) is promising for improving EOC prognosis (4, 5). Bevacizumab (BEV) can improve overall response rates in front-line or relapse therapy settings (5, 6). Front-line chemotherapy with BEV throughout improved progression-free survival (PFS) in the GOG-218 (7) and ICON7 (8) trials and prolonged overall survival (OS) in GOG-218 stage IV and ICON7-defined high-risk patients (9, 10). In total, 52 and 39% reductions in the risk of progression or death by adding BEV to chemotherapy were observed in platinum-sensitive recurrent EOC in the OCEANS (11) and GOG-213 (12) trials, respectively. A 52% reduction in the risk of progression or death was seen in platinum-resistant recurrent EOC in AURELIA (13). However, the secondary endpoint in extending OS was not achieved (9, 11–13).

BEV-related adverse effects (AEs) are well understood (7, 8, 11–14). Patients treated with BEV experienced a higher incidence of hypertension, proteinuria, gastro-intestinal (GI) and non-central nervous system (non-CNS) bleeding events, and thromboembolism (TE) than those not treated with BEV.

Based on these large clinical trials, BEV is used as the standard of care in EOC patients in many countries. However, the medical cost of adding BEV to chemotherapy may make this treatment unaffordable for EOC patients if it is not covered by the national health insurance (NHI). Moreover, the disease status in real-world patients is much more complex than that in clinical trials. Some real-world investigations have focused on the impact on PFS and AEs when BEV is added to front-line treatment (15–17). More real-world data to compare BEV effects on prognosis in recurrent EOCs or AEs between patients with or without prior BEV treatment are needed to assess cost-effectiveness (18–20).

Therefore, our study focused on EOC/tubal cancer (TC)/primary peritoneal cancer (PPC) patients who underwent front-line paclitaxel-carboplatin chemotherapy ± BEV or those with recurrent/persistent disease who underwent BEV ± chemotherapy. Our primary aim was to describe the correlation between clinico-pathological factors and physician-patient shared decision-making (SDM), as well as the survival benefits of adding BEV to front-line chemotherapy. Secondarily, we aimed to determine the safety of combination regimens of BEV plus chemotherapy, with or without prior BEV use. We aimed to identify advanced-stage or high-risk patients who may have a more favorable outcome in the front-line setting and predict which subgroup has a better prognosis after adding BEV.

## Patients and Methods

### Participants

**Figure 1:**
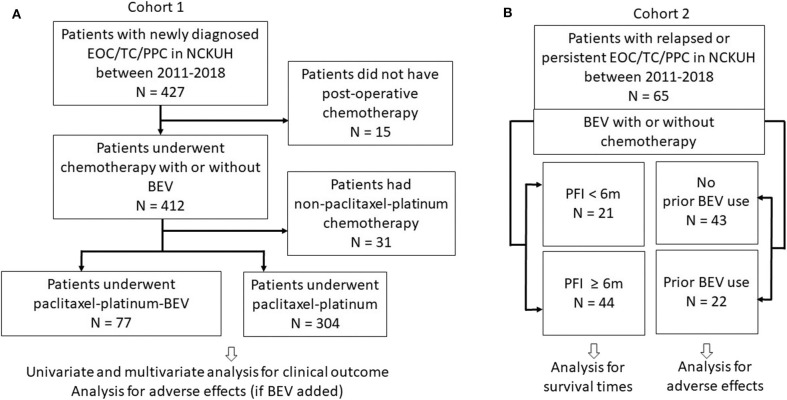
Study flow chart. **(A)** Cohort 1 patients underwent first-line paclitaxel-carboplatin chemotherapy with or without bevacizumab (BEV). **(B)** Cohort 2 patients with relapsed or persistent disease treated with BEV ± chemotherapy. Patients were also categorized as groups with progression-free interval (PFI) <6 and ≥6 m.

The clinical research protocol was approved by the National Cheng Kung University Hospital Institutional Review Board (Protocol No. A-ER-108-119). The study was performed in accordance with the Declaration of Helsinki. The requirement for informed consent was waived due to the retrospective nature of the study and the difficulties to access to patients. The study flow chart regarding patient inclusion is illustrated in Figure 1. Patients with histologically proven EOC/TC/PPC were considered eligible if they received front-line platinum-based chemotherapy as preoperative (neoadjuvant) or postoperative (adjuvant) therapy (Cohort 1) or if their relapsed diseases were treated with BEV ± any chemotherapy (Cohort 2) between January 1, 2011 and August 31, 2019.

### Study Methods

Medical records were reviewed for patients' clinical characteristics, treatment-related AEs, and treatment outcomes. Patients without postoperative front-line chemotherapy owing to any reason and those who had non-paclitaxel-platinum doublet were excluded. Cancer stage was determined according to the criteria of the International Federation of Gynecology and Obstetrics (FIGO). Residual nodules <1 cm and ≥1 cm were categorized as “optimal” and “suboptimal,” respectively. Cancer progression was defined based on the objective Response Evaluation Criteria in Solid Tumors 1.1 or the Gynecologic Cancer InterGroup (GCIG) definition. Our high-risk patients for progression was defined as FIGO stage IV, inoperable or macroscopic residuum >1 cm FIGO stage III disease, based on ICON7 definition (10). The last record was retrieved on August 31, 2019. AE severity was graded according to the Common Terminology Criteria for Adverse Events, version 4.03. OS was calculated based on the diagnosis date. PFS and PFI were determined using the date of last contact or progression after front-line chemotherapy. PFI <6 months (m) and ≥6 m were categorized as “platinum-resistant” and “platinum-sensitive,” respectively. “PFS2-PFS” was calculated based on the date of the first relapse or persistence to the next progression or death.

### Statistical Analysis

Data were analyzed using SPSS (version 19.0; SPSS Inc., Chicago, IL, USA) (21). Interval variables are presented as means ± standard deviations. Differences between groups were tested using the Mann–Whitney U test. Frequency distributions between categorical variables were compared using Pearson *X*^2^ or Fisher's exact tests. Survival was estimated using the Kaplan–Meier method and compared using log-rank tests. P < 0.05 (two-sided) was considered statistically significant. Cox proportional hazards models were used to estimate hazard ratios (HRs) and confidence intervals (CIs). Possible confounders were included in multivariate analyses. The independent effect of BEV use on survival and disease progression was analyzed in the multivariate analysis.

## Results

### Patient Demographics

#### Cohort 1 Patients

Newly diagnosed patients (*n* = 381) aged 21–88 years (y) (mean, 53.6 ± 11.4 y) were enrolled. Only 37 patients were aged >70 y. Most patients (*n* = 359, 94.2%) were diagnosed with EOC; eight (2.1%) were diagnosed with TC and 14 (3.7%) with PPC. Of these, 77 (20.2%) received postoperative paclitaxel-carboplatin doublet plus BEV. A total of 147 (38.6%) were classified as having FIGO stages I–II and 234 (61.4%) as having stages III–IV; 116 (30.4%) patients were in the high-risk subgroup. More than half of the patients (*n* = 203, 53.3%) had serous histology, 108 (28.3%) had clear cell carcinoma (CCC) histology, 39 (10.2%) had endometrioid histology, and 20 (5.2%) had mucinous histology. The other histological types included transitional cell carcinoma and poorly-differentiated adenocarcinoma. The median follow-up period was 33.7 m (range 1.7–99.9 m).

BEV dosage was 7.5–15.1 (mean, 9.2 ± 1.5) mg/kg, and number of treatment cycles was 1–17 (mean 7.9 ± 5.0). The dosage was <10 mg/kg/cycle in 71.4% of patients, and <10 cycles were used in 76.6% of patients. BEV dosages were similar between stage III–IV (*n* = 67) and the high-risk patients (*n* = 37) (mean, 9.3 ± 1.6 and 9.3 ± 1.8 mg/kg, respectively). There was no difference in the number of treatment cycles between these groups (mean, 7.9 ± 5.0 and 7.9 ± 4.6 cycles, respectively). During follow-up, 45 patients (58.4%) in the chemotherapy plus BEV group developed progressive disease, and 22 (28.6%) died; 156 patients (51.3%) in the chemotherapy alone group had progressive disease, and 114 patients (37.5%) died.

Relationships between whether BEV was added to chemotherapy and clinicopathological factors are presented in Table 1. Women who received chemotherapy plus BEV, had significantly more advanced stage (87.0 vs. 54.9%), serous histology (66.2 vs. 50.0%), preoperative chemotherapy (9.1 vs. 1.6%), residual tumor size ≥1 cm (32.5 vs. 19.7%), and triweekly delivery of postoperative chemotherapy (89.6 vs. 71.1%), when compared with patients treated with chemotherapy alone.

**Table 1:** Clinico-pathological characteristics of newly diagnosed patients (Cohort 1) (*N* = 381).

	**Chemotherapy Alone *N* = 304**	**Chemotherapy plus bevacizumab *N* = 77**	***P***
Age (mean ± SD)	53.6 ± 11.0	53.5 ± 11.0	0.850
Cancer origin			0.315
Ovary	289 (95.1)	70 (90.9)	
Tube	6 (2.0)	2 (2.6)	
Peritoneum	9 (3.0)	5 (6.5)	
Stage			<0.001
Early	137 (45.1)	10 (13.0)	
I	98 (32.3)	6 (7.8)	
II	39 (12.8)	4 (5.2)	
Advanced	167 (54.9)	67 (87.0)	
III	135 (44.4)	44 (57.1)	
IV	32 (10.5)	23 (29.9)	
Histology			0.013
Serous	152 (50.0)	51 (66.2)	
Non-serous	152 (50.0)	26 (33.8)	
Clear cell	91 (29.9)	17 (22.1)	
Endometrioid	34 (11.2)	5 (6.5)	
Mucinous	18 (5.9)	2 (2.6)	
Other type	9 (3.0)	2 (2.6)	
Tumor grade			0.143
Low (grade 1)	11 (3.6)	3 (3.9)	
High (grade 2 or 3)	284 (93.4)	73 (94.8)	
Unspecified	9 (3.0)	1 (1.3)	
Preoperative chemotherapy			0.003
No	299 (98.4)	70 (90.9)	
Yes	5 (1.6)	7 (9.1)	
Cytoreduction			0.046
Optimal	235 (77.3)	49 (63.6)	
Suboptimal	60 (19.7)	25 (32.5)	
Unknown	9 (3.0)	3 (3.9)	
Postoperative chemotherapy			0.001
Triweekly	216 (71.1)	69 (89.6)	
Dose dense	88 (28.9)	8 (10.4)	
Bevacizumab exposure			
Mean No. of cycles (range)	–	7.9 (1–17)	–
Mean dose (mg/kg) (range)	–	9.2 (7.5–15.1)	
PFI			0.354
≥12 months (platinum-sensitive)	160 (52.6)	33 (42.9)	
6–12 months (partially platinum-sensitive)	54 (17.8)	17 (22.1)	
<6 months (platinum-resistant)	90 (29.6)	27 (35.1)	
Progression	156 (51.3)	45 (58.4)	
Death	114 (37.5)	22 (28.6)	

*Chemotherapy regimen was referred to paclitaxel-carboplatin doublets*.

*Data was analyzed by X^2^ or Fisher's exact test*.

*SD, standard deviation; PFI, progression-free interval*.

#### Cohort 2 Patients

Sixty-five patients with relapsed or persistent disease (mean age, 52.5 ± 11.0 y; range, 18–74 y) were enrolled. Patients' baseline characteristics are shown in Table 2. Of those treated with various chemotherapy regimens, 39 (60.0%) received adjuvant platinum-based chemotherapy and 22 (33.8%) had prior BEV use. BEV dosage was 7.0–12.6 (mean, 9.5 ± 1.3) mg/kg and 7.9–11.6 (mean, 10.2 ± 1.1) mg/kg for patients with the first and ≥2 relapses, respectively; the number of treatment cycles was 1–22 (mean 5.3 ± 3.1) and 2–19 (mean 6.5 ± 4.4), respectively. The median follow-up period was 3.6 m (range 0.1–37.3 m). Most patients (*n* = 60, 92.3%) were diagnosed with EOC; 2 (3.1%) and 3 (4.6%) were diagnosed with TC and PPC, respectively. Thirty-nine patients (60.0%) had serous histology, 10 (15.4%) had CCC histology, 6 (9.2%) had endometrioid histology, and 4 (6.2%) had mucinous histology. The other histological types included transitional cell carcinoma and poorly-differentiated adenocarcinoma. During follow-up, 39 patients (60.0%) developed progressive disease, and 29 (44.86%) died.

**Table 2:** Clinico-pathological characteristics of patients with recurrent or persistent disease (Cohort 2) (*N* = 65).

	**Platinum-resistant (PFI < 6 m) *N* = 21**	**Platinum-sensitive (PFI ≥ 6 m) *N* = 44**	***P***
Age (mean ± SD)	53.7 ± 7.8	51.9 ± 12.2	0.536
Cancer origin			0.682
Ovary	10 (95.2)	40 (91.0)	
Tube	–	2 (4.5)	
Peritoneum	1 (4.8)	2 (4.5)	
Stage			
I	–	5 (11.4)	–
II	1 (4.8)	4 (9.1)	
III	12 (57.1)	28 (63.6)	
IV	8 (38.1)	7 (15.9)	
Histology			0.386
Serous	11 (52.4)	28 (63.6)	
Non-serous	10 (47.6)	16 (36.4)	
Clear cell	–	2 (4.5)	
Endometrioid	2 (9.5)	4 (9.1)	
Mucinous	8 (38.1)	4 (9.1)	
Other type	–	6 (13.6)	
Tumor grade			–
Low (grade 1)	–	2 (4.5)	
High (grade 2 or 3)	21 (100)	40 (91.0)	
Unknown	–	2 (4.5)	
Primary cytoreduction			0.568
Optimal	13 (61.9)	31 (70.5)	
Suboptimal	7 (33.3)	12 (27.3)	
Unknown	1 (4.8)	1 (2.3)	
PFI after first-line chemotherapy			–
<6 months	21 (100)	–	
6–12 months	–	21 (47.7)	
≥12 months	–	23 (52.3)	
Chemotherapy regimen			
PTX-platinum (Triweekly)	3 (14.3)	11 (25.0)	
PTX-platinum (Dose dense)	2 (9.5)	5 (11.4)	
PLD	8 (38.1)	3 (6.8)	
PLD–platinum	6 (28.6)	16 (36.4)	
GEM (Dose dense)	–	2 (4.5)	
GEM–platinum	1 (4.8)	2 (4.5)	
Topotecan	–	3 (6.8)	
Topotecan (Dose dense)	1 (4.8)	–	
Cyclophosphamide–platinum	–	1 (2.3)	
Next progression			0.194
No	6 (28.6)	20 (45.5)	
Yes	15 (71.4)	24 (54.5)	

*PFI, progression-free interval; PTX, paclitaxel; PLD, pegylated liposomal doxorubicin; GEM, gemcitabine*.

Relationships between PFI <6 or ≥6 m and clinicopathological factors are presented in Table 2. No statistical difference between PFI of 6 m and stage, histology, tumor grade, residual tumor size during primary surgery, or next disease progression was observed.

### Clinical Outcomes

#### Cohort 1 Patients With Advanced-Stage Disease

**Figure 2:**
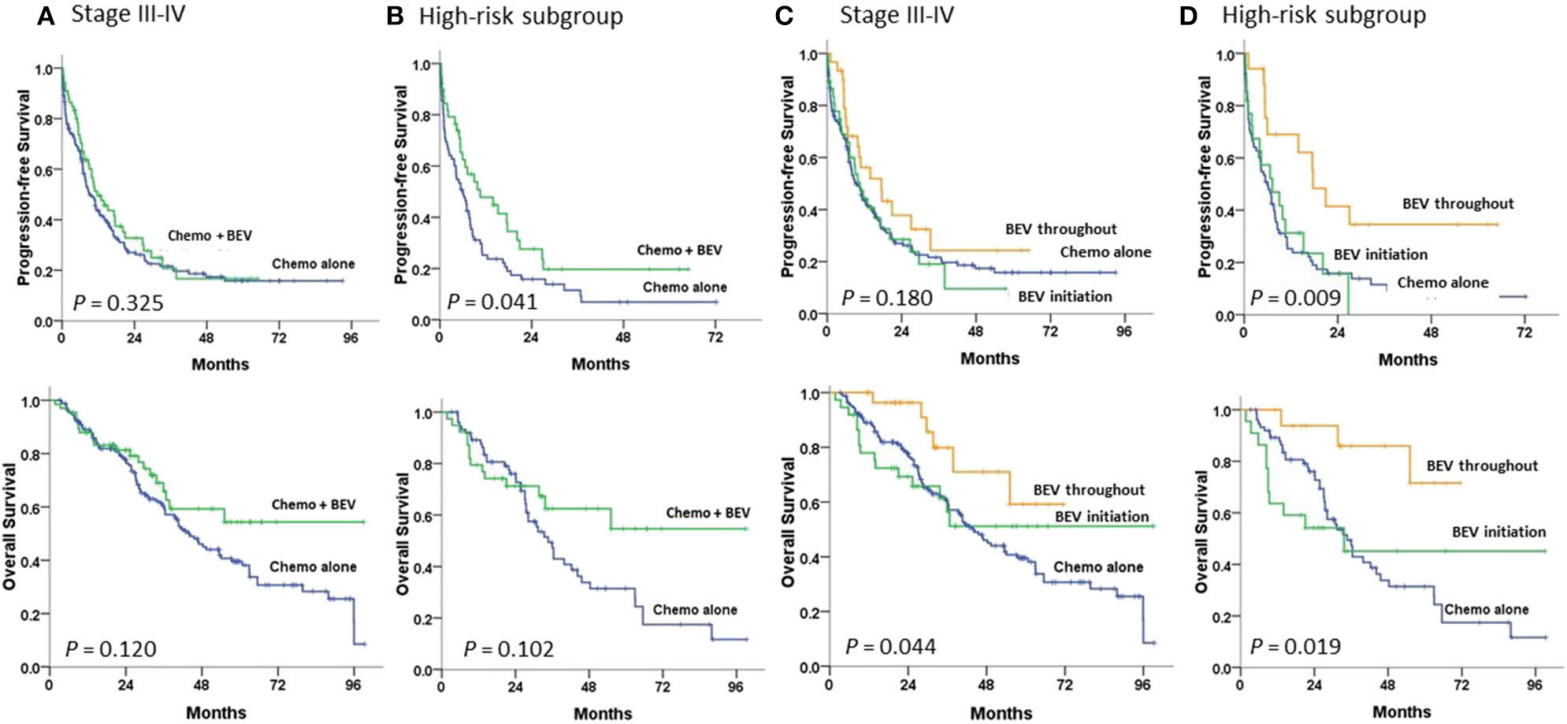
Progression-free and overall survival in **(A,C)** stage III–IV patients and **(B,D)** high-risk patients undergoing first-line therapy, tested by the log-rank test. **(A,B)** between chemotherapy (Chemo) with and without bevacizumab (BEV); **(C,D)** between Chemo with BEV throughout, BEV initiation, and Chemo alone.

Survival curves for 234 patients stratified by means of BEV added to chemotherapy or chemotherapy alone are illustrated in Figure 2. The median PFS duration was greater in the subgroup with chemotherapy plus BEV than with chemotherapy alone (11.6 vs. 9.3 m, *P* = 0.325; HR 0.84, 95% CI, 0.6–1.19) among advanced-stage patients (Figure 2A), similar to that found among the high-risk patients (10.5 vs. 6.0 m, *P* = 0.035; HR 0.62, 95% CI, 0.39–0.97) (Figure 2B). The median OS was not achieved in the subgroup with BEV and was 43.7 m in those without BEV (*P* = 0.123; HR 0.69, 95% CI, 0.43–1.11) among advanced-stage patients (Figure 2A); similar results were seen among the high-risk patients (not reached versus 34.7 m, *P* = 0.101; HR 0.61, 95% CI, 0.33–1.10) (Figure 2B).

Compared to patients with chemotherapy alone, among the high-risk patients, patients with BEV throughout had a significantly greater PFS (17.6 m; *P* = 0.005; HR 0.39, 95% CI, 0.20–0.75) and OS (not reached; *P* = 0.010; HR 0.22, 95% CI, 0.07–0.72) (Figure 2D). However, among advanced-stage patients, patients with BEV throughout had a significantly greater OS (not reached; *P* = 0.030; HR 0.41, 95% CI, 0.18–0.93), but not PFS, than those with chemotherapy alone (Figure 2C).

**Figure 3:**
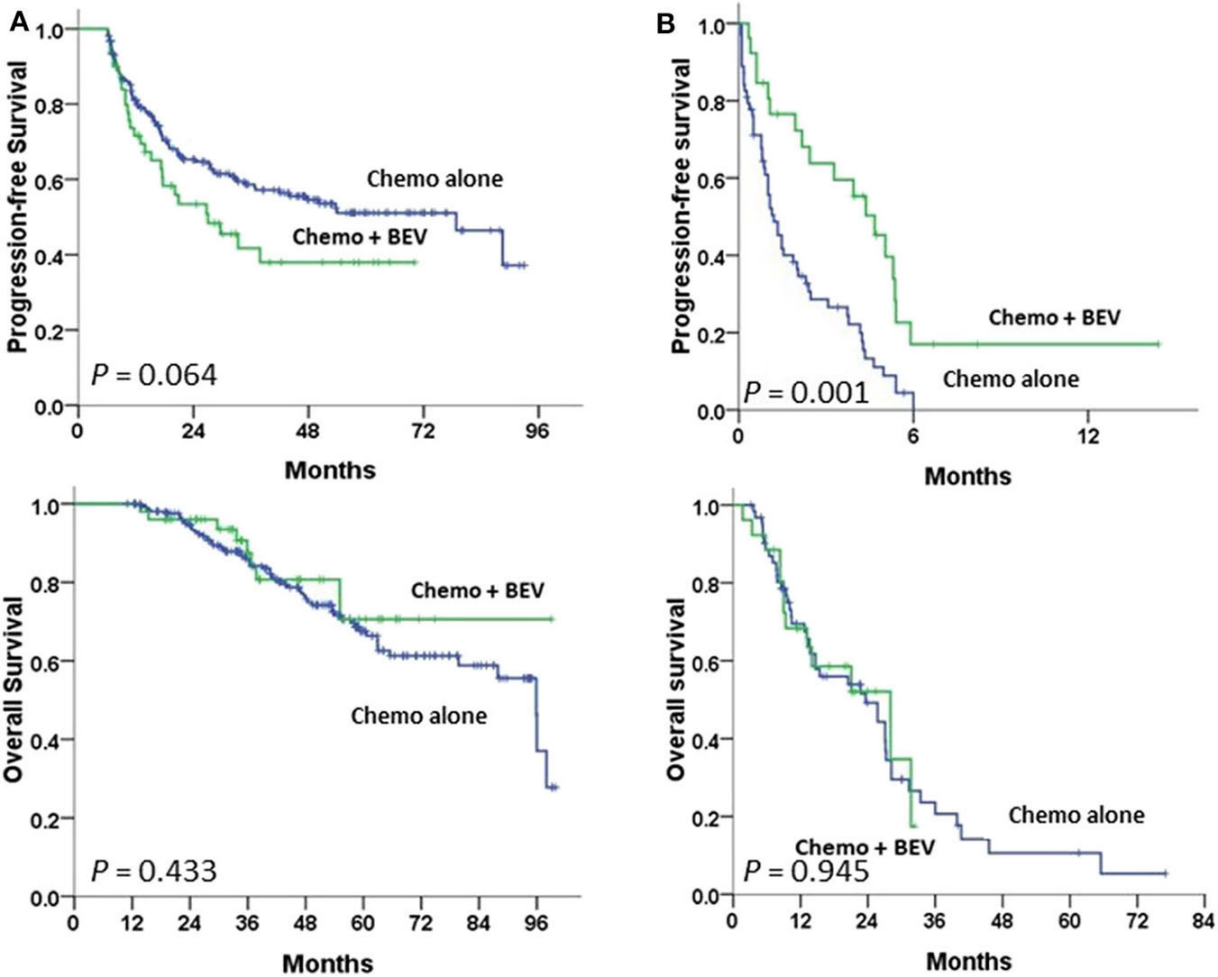
Progression-free and overall survival between paclitaxel-carboplatin chemotherapy (Chemo) with and without bevacizumab (BEV) in stage III–IV patients undergoing first-line therapy were tested using the log-rank test **(A)** in the progression-free interval (PFI) ≥6 m subgroup and **(B)** in the PFI <6 m subgroup.

Advanced-stage patients treated with chemotherapy plus BEV in the subgroup of PFI <6 m had a significantly greater PFS (*P* = 0.001), but not OS (Figure 3B) when compared to those treated with chemotherapy alone; no statistical difference in PFS or OS was observed in the subgroup of PFI ≥ 6 m (Figure 3A). No statistical difference in the impacts of BEV on PFS or OS between clear cell and serous histology was observed in the early or advanced stage (Supplementary Figure 1).

#### Cohort 2 With Relapsed or Persistent Disease

**Figure 4:**
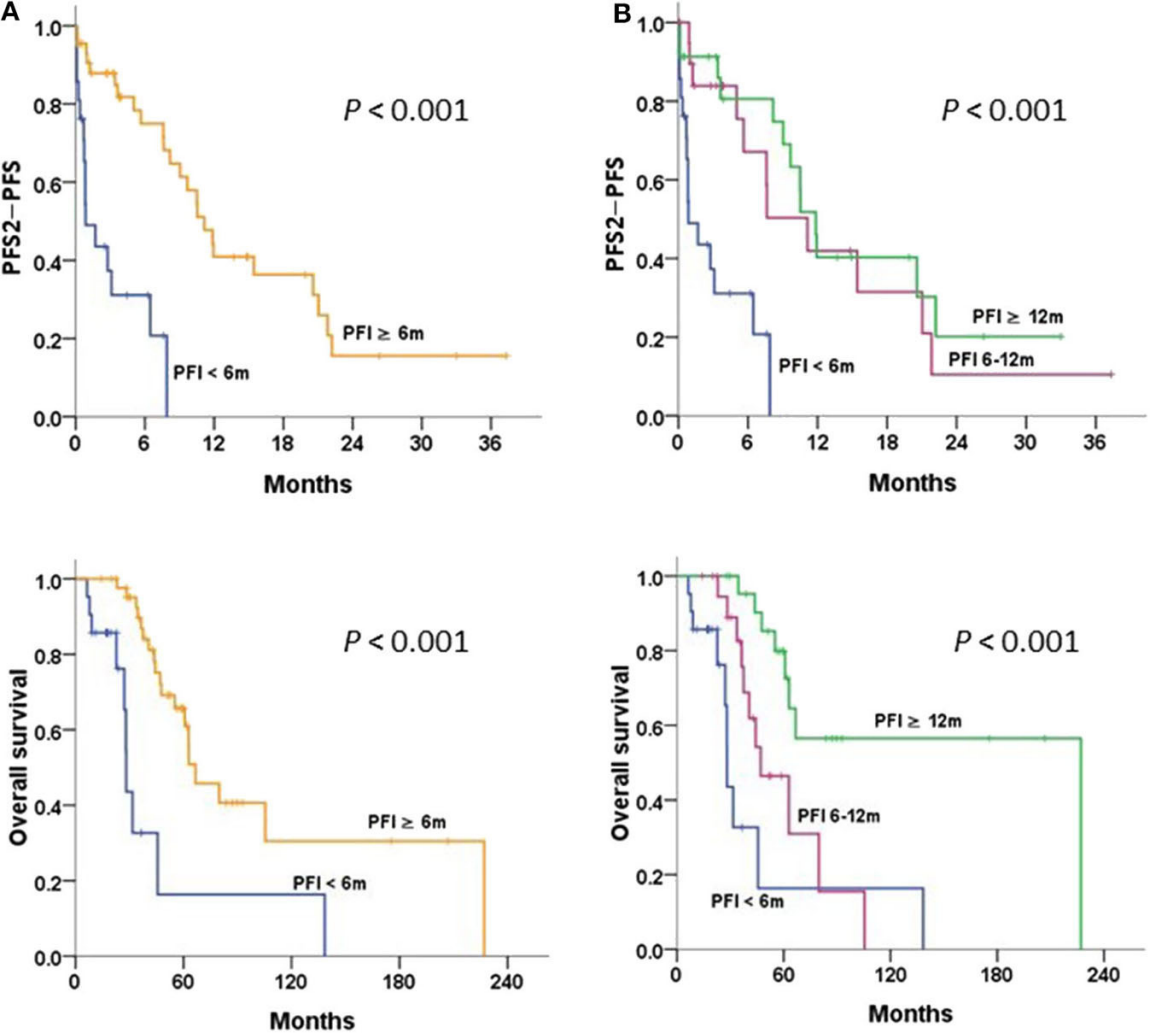
Progression-free survival 2 (PFS2)-PFS and overall survival in patients with relapsed/persistent disease treated with bevacizumab (BEV) ± chemotherapy (Chemo), stratified by **(A)** progression-free interval (PFI) < and ≥6 m; **(B)** PFI <6, 6–12, and ≥12 m, tested by the log-rank test.

The survival curves stratified by PFI of 6 and 12 m are illustrated in Figure 4. Patients with PFI ≥6 m after primary therapy had a significantly better OS and PFS2-PFS than those with PFI <6 m (*P* < 0.001 and *P* < 0.001, respectively) (Figure 4A). Patients with a longer PFI had more favorable survival. Patients with PFI ≥12 m had greater OS and PFS2-PFS than those with PFI <6 m (*P* < 0.001 and *P* < 0.001, respectively) (Figure 4B).

### Univariate and Multivariate Analyses

In the univariate analysis, residual nodules ≥1 cm and PFI <6 m were significantly associated with a high risk of death and disease progression in Cohort 1 patients with advanced disease Table 3. Age ≥53 years was associated with a high risk of death due to cancer. In a multivariate-adjusted model, PFI <6 m was the strongest predictor of disease progression (HR 27.78, 95% CI 15.87–47.62) and death (HR 5.92, 95% CI 3.82–9.17) (Table 4). BEV throughout and optimal cytoreduction in the front-line setting were independent predictors of reduced cancer progression (HR 0.43, 95% CI 0.25–0.73, and HR 0.63, 95% CI 0.44–0.90, respectively). Although BEV throughout was a predictor of cancer death, its impact was influenced by confounders in the multivariate analysis.

**Table 3:** Univariate analysis in newly diagnosed cancer patients with stage III and IV disease (*n* = 234).

**Univariate analysis**							
**Variable**	**N**	**Progression events**	**Median time to progression (months)**	**HR for progression (95% CI)**	**Death events**	**Median time to death (months)**	**HR for death (95% CI)**
Age (years)							
<53	99	78	9.8	1.00	40	55.1	1.00
≥53	135	88	10.8	0.87 (0.64–1.18)	71	39.9	1.51 (1.02–2.23)
Histology							
Non-serous	57	40	7.0	1.00	25	36.3	1.00
Serous	177	126	11.07	0.84 (0.54–1.32)	86	47.0	0.78 (0.55–1.12)
Tumor grade							
Low	7	3	9.80	1.00	0	–	1.00
High	227	162	10.5	1.78 (0.57–5.60)	111	33.2	21.32 (0.21–2146.94)
Pre-operative chemotherapy							
No	223	161	10.5	1.00	106	47.0	1.00
Yes	11	5	17.0	0.74 (0.30–1.80)	5	22.1	1.83 (0.74–4.52)
Cytoreduction							
Optimal	150	97	15.7	1.00	65	54.1	1.00
Suboptimal	84	69	5.4	2.25 (1.65–3.08)	46	33.7	1.77 (1.21–2.60)
Post-operative chemotherapy							
Triweekly	163	114	10.3	1.00	66	46.7	1.00
Dose dense	71	52	11.0	0.93 (0.67–1.29)	45	45.6	1.17 (0.80–1.72)
PFI							
≥6 months	145	94	17.6	1.00	55	63.0	1.00
<6 months	89	72	1.9	21.48 (13.64–33.85)	56	23.6	6.11 (4.07–9.17)
Bevacizumab							
No	167	122	9.3	1.00	90	43.7	1.00
Initiation	37	26	10.5	0.99 (0.65–1.53)	15	–	0.95 (0.55–1.64)
Throughout	30	18	17.5	0.69 (0.42–1.13)	6	–	0.41 (0.18–0.93)

*Chemotherapy regimen was referred to paclitaxel-carboplatin doublets*.

*HR, hazard ratio; PFI, progression-free interval*.

**Table 4:** Multivariate analysis in newly diagnosed cancer patients with stage III and IV disease (*n* = 234).

**Multivariate analysis**		
**Variable**	**HR for progression (95% CI)**	**HR for death (95% CI)**
Age (≥ 53 vs <53 years)	0.98 (0.71–1.37)	1.45 (0.96–2.19)
Histology (serous vs non-serous)	0.96 (0.63–1.46)	1.17 (0.73–1.89)
Cytoreduction (suboptimal vs optimal)	1.58 (1.11–2.26)	1.08 (0.72–1.62)
Post-operative chemotherapy (dose dense vs triweekly)	1.03 (0.72–1.46)	0.86 (0.57–1.30)
PFI (<6 vs ≥ 6 months)	27.78 (15.87–47.62)	5.92 (3.82–9.17)
Bevacizumab (throughout vs no)	0.43 (0.25–0.73)	0.43 (0.19–1.02)

*Chemotherapy regimen was referred to paclitaxel-carboplatin doublets*.

*HR, hazard ratio; PFI, progression-free interval*.

By cox proportional analysis in Cohort 2 patients, those with PFI of ≥6 m after primary therapy had significantly lower risks of death (HR 0.22, 95% CI 0.10–0.50) and next progression (HR 0.17, 95% CI 0.08–0.39) than those with PFI <6 m.

### Safety Assessment

In total, 142 (77 Cohort 1 and 65 Cohort 2) patients were assessed for AEs (Table 5). Most AEs were mild (≤ grade 2), treatable, and reversible. Commonly observed AEs ≥ grade 3 included neutropenia (*n* = 44, 31.0%), febrile neutropenia (*n* = 12, 8.4%), anemia (*n* = 6, 4.2%), diarrhea (*n* = 2, 1.4%), and anorexia (*n* = 1, 0.7%). BEV-associated AEs were not observed. All cases of non-CNS bleeding (*n* = 26, 3.5%), hypertension (*n* = 21, 14.8%), proteinuria (*n* = 13, 9.2%), or venous thromboembolic events (*n* = 3, 2.1%) were rare and treatable in patients with or without prior BEV use.

**Table 5:** Adverse effects and grades in ovarian/tubal/peritoneal patients treated with bevacizumab plus chemotherapy (*N* = 142).

	**Front-line therapy with bevacizumab** ***n*** **=** **77**				**Relapse therapy without prior bevacizumab** ***n*** **=** **43**				**Relapse therapy with prior bevacizumab** ***n*** **=** **22**			
**Adverse effects**	**Grade**				**Grade**				**Grade**			
	**1**	**2**	**3**	**4**	**1**	**2**	**3**	**4**	**1**	**2**	**3**	**4**
Bleeding (non-CNS)												
Nasal	8 (10.4)	–	–	–	4 (9.3)	–	–	–	2 (9.1)	–	–	–
Gingival	3 (3.9)	–	–	–	–	–	–	–	2 (9.1)	–	–	–
Conjunctival	–	–	–	–	1 (2.3)	–	–	–	–	–	–	–
Gastrointestinal	3 (3.9)	–	–	–	1 (2.3)	1 (2.3)	–	–	1 (4.5)	–	–	–
Hypertension	7 (9.1)	3 (3.9)	4 (5.2)	–	1 (2.3)	4 (9.3)	–	–	1 (4.5)	1 (4.5)	–	–
Proteinuria	10 (13.0)	–	–	–	3 (7.0)	–	–	–	–	–	–	–
Wound disruption	4 (5.2)	–	–	–	–	–	–	–	1 (4.5)	–	–	–
Venous thromboembolism	2 (2.6)	–	–	–	–	1 (2.3)	–	–	–	–	–	–
Febrile neutropenia	–	–	7 (9.1)	–	–	–	3 (7.0)	–	–	–	2 (9.1)	–
Neutropenia	19 (24.7)	7 (9.1)	28 (36.4)	1 (1.3)	15 (34.9)	1 (2.3)	8 (18.6)	1 (2.3)	6 (27.3)	3 (13.6)	6 (27.3)	–
Anemia	34 (44.2)	18 (23.4)	3 (3.9)	–	16 (37.2)	10 (23.3)	3 (7.0)	–	8 (36.4)	5 (22.7)	–	–
Nausea	36 (46.8)	2 (2.6)	–	–	20 (46.5)	2 (4.7)	–	–	9 (40.9)	–	–	–
Vomiting	12 (15.6)	2 (2.6)	–	–	11 (25.6)	4 (9.3)	–	–	2 (9.1)	–	–	–
Mucositis	18 (23.4)	–	–	–	15 (34.9)	4 (9.3)	–	–	6 (27.3)	–	–	–
Diarrhea	7 (9.1)	2 (2.6)	1 (1.3)	–	4 (9.3)	–	–	–	3 (13.6)	1 (4.5)	1 (4.5)	–
Constipation	35 (45.5)	3 (3.9)	–	–	14 (32.6)	–	–	–	13 (59.1)	1 (4.5)	–	–
Anorexia	32 (41.6)	2 (2.6)	1 (1.3)	–	14 (32.6)	3 (7.0)	–	–	11 (50.0)	1 (4.5)	–	–
Paresthesia	45 (58.4)	22 (28.6)	–	–	23 (53.5)	8 (18.6)	–	–	12 (54.5)	9 (40.9)	–	–
Alopecia	2 (2.6)	62 (80.5)	–	–	5 (11.6)	29 (67.4)	–	–	1 (4.5)	18 (81.8)	–	–

*Data was shown as n (%)*.

*Grades of adverse effects were assessed according to the Common Terminology Criteria for Adverse Events*.

*CNS, central nervous system*.

*Nasal bleeding occurred after 2 to 5 cycles of chemotherapy plus bevacizumab*.

*The following adverse effects did not occur in our participants: Left ventricular systolic dysfunction, congestive heart failure, fistula/abscess, gastrointestinal perforation, reversible posterior leukoencephalopathy syndrome, or arterial thromboembolism*.

## Discussion

We found improved PFS as a benefit of BEV throughout triweekly in the dosage range of 7.5–15 mg/kg, which is consistent with results of independent phase 3 trials assessing front-line therapy (7, 8) and real-world data (15–17). PFI <6 m independently predicted shorter PFS and OS, but BEV may reduce platinum-resistant relapse; these findings are in concordance with the prospective single-arm results in JGOG3022 (17). Moreover, to our knowledge, this is the first real-world study to provide data on improved OS as an advantage of BEV throughout, relative to chemotherapy alone, in front-line therapy in advanced-stage EOC patients and the high-risk subgroup.

The GCIG considered PFS as the primary endpoint in front-line therapy for ovarian cancer, and not OS, because of the confounding effects of post-progression therapy on OS (7, 22). Advances in various post-progression therapies, such as BEV (11–14, 18), trientine (23), and poly(adenosine diphosphate [ADP]-ribose) polymerase (PARP) inhibitor (4, 24–26), may help extend patients' lives. We provided evidence while we observed a longer median OS in the BEV throughout group compared to that in GOG-218 and ICON7, even in patients who received only chemotherapy. When adjusted by multivariate analysis, our real-world findings corresponded to the GCIG consensus and emphasized the role of BEV throughout in PFS prolongation. This phenomenon is observed in the improved OS after using BEV in relapsed EOC, specifically in the platinum-sensitive subgroup.

A significantly improved PFS when adding olaparib after a response to chemotherapy plus BEV has occurred and a higher incidence of reversible grade ≥ 3 haematologic AEs were reported in the PAOLA-1 trial (27). Tewari et al. reported the reductions in risk of death for BRCA1/2-mutated or non-BRCA1/2 homologous recombination deficient (HRD) carcinomas when compared to the wild type (9). However, BRCA/HRD testing was not predictive of BEV activity. Our patients had a shorter median PFS than that of the non-Olaparib arm in PAOLA-1 trial (27) or those in real-world studies (15–17). The reason for these discrepancies may be multifactorial, including different study designs, shorter maintenance duration or lower dosage of BEV, more complex disease, or reimbursement issues in the real-world setting.

Platinum resistance is an indicator of poor prognosis, and its involved mechanism is complex (28–30). Lee et al. reported that the effectiveness of BEV-included chemotherapy was feasible but varied according to the chemotherapy partner in platinum-resistant EOC (20). A real-world small study showed PFS benefits of early BEV-added chemotherapy, but insignificant survival differences between platinum-resistant and -sensitive recurrent EOC (31). However, we provided more favorable oncologic outcomes of BEV-added relapse therapy in platinum-sensitive patients. Furthermore, CCC has been thought of as a platinum-resistant malignancy. The histological distribution of EOC in Asia is quite different from that in the West. Serous, endometrioid, and CCC histology constituted 40–50, 15–20, and 15–20%, respectively, of EOC in Taiwan from 2000–2008 (32). Early-stage patients with CCC or grade 3 tumor were included in ICON7 trial as a subgroup of particular interest (8). Our early-stage patients used BEV following ICON7 inclusion criteria. Komiyama et al. reported that BEV-added front-line therapy was effective for advanced-stage clear cell carcinoma (17). However, the lack of benefits of BEV on clinical outcomes across different histologies in our study is similar to findings recorded in the ICON7 final results (10). Therefore, BEV could be applied regardless of histology in either an early or advanced stage.

Haematologic AEs were the most common in BEV-included trials in EOC (7, 8, 11–14), while similar results were observed in our study. Its safety was also promising in our relapse therapy. The cumulative incidences of hypertension and proteinuria were associated with median cumulative BEV dosages (33) or the treatment durations (17) in Asian women. However, our patients had less BEV-specific incidences and lower grades of AEs. This may be related to lower dosages, or fewer treatment cycles in most patients, which were in concordance with lower incidence rates of hypertension, proteinuria, GI, or non-CNS bleeding events in the BEV throughout arm in ICON7 than that in GOG-218 (7, 8).

A study carried out in Belgium reported the relatively high cost-effectiveness of BEV with the most promising results in front-line treatment of stage IV EOC patients (34). The prognostic factors, e.g., advanced stage, histology, pre- and post-operative chemotherapy, and residual tumor size, were adjusted for during multivariate analysis, but expert-patient SDM regarding BEV utilization may possibly be influenced by choices of post-progression therapy or socio-economic factors which were not included in our analysis.

There are some other limitations to our study. BEV and BRCA/HRD testing has not yet been covered by our NHI. Hence, we are unable to provide real-world data regarding BEV in patients with BRCA mutation/HRD. Although most AEs were mild and treatable, the number of patients treated with BEV after prior BEV is too small to draw a conclusion in our relapsed cohort. Further research should be conducted to clarify these questions.

In conclusion, adding BEV to traditional front-line or relapse therapy was safe in EOC. This strategy as maintenance therapy was effective in extending PFS and OS in advanced-stage patients. Platinum-sensitivity was the strongest prognostic factor. Platinum-sensitive relapsed EOC patients treated with BEV-added chemotherapy had a significant improvement in OS and had a longer duration in progressing to the next progression.

## Data Availability Statement

The datasets analyzed in this article are not publicly available because they contain information that could compromise research participant privacy. Requests to access the datasets should be directed to Yu-Fang Huang, yufangh@mail.ncku.edu.tw.

## Ethics Statement

The clinical research protocol was approved by the National Cheng Kung University Hospital Institutional Review Board (Protocol No. A-ER-108-119). The study was performed in accordance with the Declaration of Helsinki. The requirement for informed consent was waived due to the retrospective nature of the study and the difficulties to access to patients.

## Author Contributions

All authors participated in the conception or design of the present study. P-YW, Y-MC, M-RS, Y-FH, and C-YC conducted research. P-YW and Y-MC acquired data. Y-CC and Y-FH performed the statistical analysis and P-YW, Y-FH, and C-YC interpreted the results. M-RS and C-YC provided administrative support. All authors drafted and revised the article for important intellectual content and have approved the final submitted version of the manuscript.

## Conflict of Interest

The authors declare that the research was conducted in the absence of any commercial or financial relationships that could be construed as a potential conflict of interest.

## References

[1] Siegel RL Miller KD Jemal A Cancer statistics, 2018. CA Cancer J Clin. (2018) 68:7–30. 10.3322/caac.21442 29313949

[2] Ferlay J Colombet M Soerjomataram I Mathers C Parkin DM Piñeros M. Estimating the global cancer incidence and mortality in 2018: GLOBOCAN sources and methods. Int J Cancer. (2019) 144:1941–53. 10.1002/ijc.31937 30350310

[3] Chang LC Huang CF Lai MS Shen LJ Wu FL Cheng WF. Prognostic factors in epithelial ovarian cancer: a population-based study. PLoS ONE. (2018) 13:e0194993. 10.1371/journal.pone.0194993 29579127 PMC5868839

[4] Franzese E Centonze S Diana A Carlino F Guerrera LP Di Napoli M. PARP inhibitors in ovarian cancer. Cancer Treat Rev. (2019) 73:1–9. 10.1016/j.ctrv.2018.12.002 30543930

[5] Jiang Y Sun X Kong B Jiang J. Antiangiogenesis therapy in ovarian cancer patients: An updated meta-analysis for 15 randomized controlled trials. Medicine. (2018) 97:e11920. 10.1097/MD.0000000000011920 30142803 PMC6112884

[6] Wu YS Shui L Shen D Chen X. Bevacizumab combined with chemotherapy for ovarian cancer: an updated systematic review and meta-analysis of randomized controlled trials. Oncotarget. (2017) 8:10703–13. 10.18632/oncotarget.12926 27793044 PMC5354693

[7] Burger RA Brady MF Bookman MA Fleming GF Monk BJ Huang H. Incorporation of bevacizumab in the primary treatment of ovarian cancer. N Engl J Med. (2011) 365:2473–83. 10.1056/NEJMoa1104390 22204724

[8] Perren TJ Swart AM Pfisterer J Ledermann JA Pujade-Lauraine E Kristensen G. A Phase 3 of bevacizumab in ovarian cancer [published correction appears. N Engl J Med. (2011) 365:2484-96. 10.1056/NEJMoa1103799 22204725

[9] Tewari KS Burger RA Enserro D Norquist BM Swisher EM Brady MF. Final overall survival of a randomized trial of bevacizumab for primary treatment of ovarian cancer. J Clin Oncol. (2019) 37:2317–28. 10.1200/JCO.19.01009 31216226 PMC6879307

[10] Oza AM Cook AD Pfisterer J Embleton A Ledermann JA Pujade-Lauraine E. Standard chemotherapy with or without bevacizumab for women with newly diagnosed ovarian cancer (ICON7): overall survival results of a phase 3 randomised trial. Lancet Oncol. (2015) 16:928–36. 10.1016/S1470-2045(15)00086-8 26115797 PMC4648090

[11] Aghajanian C Goff B Nycum LR Wang YV Husain A Blank SV Final overall survival and safety analysis of OCEANS, a phase 3 trial of chemotherapy with or without bevacizumab in patients with platinum-sensitive recurrent ovarian cancer. Gynecol Oncol. (2015) 139:10–6. 10.1016/j.ygyno.2015.08.004 26271155 PMC4993045

[12] Coleman RL Brady MF Herzog TJ Sabbatini P Armstrong DK Walker JL. Bevacizumab and paclitaxel-carboplatin chemotherapy and secondary cytoreduction in recurrent, platinum-sensitive ovarian cancer (NRG Oncology/Gynecologic Oncology Group study GOG-0213): a multicentre, open-label, randomised, phase 3 trial. Lancet Oncol. (2017) 18:779–91. 10.1016/S1470-2045(17)30279-6 28438473 PMC5715461

[13] Pujade-Lauraine E Hilpert F Weber B Reuss A Poveda A Kristensen G. Bevacizumab combined with chemotherapy for platinum-resistant recurrent ovarian cancer: The AURELIA open-label randomized phase III trial [published correction appears. J Clin Oncol. (2014) 32:1302–8. 10.1200/JCO.2013.51.4489 24637997

[14] Pignata S Lorusso D Joly F Gallo C Colombo N Sessa C Chemotherapy plus or minus bevacizumab for platinum-sensitive ovarian cancer patients recurring after a bevacizumab containing first line treatment: The randomized phase 3 trial MITO16B-MaNGO OV2B-ENGOT OV17. J Clin Oncol. (2018) 15 suppl:5506–6. 10.1200/JCO.2018.36.15_suppl.5506

[15] An Observational Study of Avastin (bevacizumab) in Combination With Carboplatin/Paclitaxel in First Line In Patients With Advanced Epithelial Ovarian Fallopian Tube or Primary Peritoneal Cancer (OTILIA) (ClinicalTrials.gov Identifier: NCT01697488). Available online at: https://clinicaltrials.gov/ct2/show/NCT01697488 (accessed February 1, 2020).

[16] Hall M Bertelli G Li L Green C Chan S Yeoh CC. Role of front-line bevacizumab in advanced ovarian cancer: the OSCAR study Int J Gynecol Cancer. (2019). [Epub ahead of print]. 10.1136/ijgc-2019-000512. 31780570

[17] Komiyama S Kato K Inokuchi Y Takano H Matsumoto T Hongo A. Bevacizumab combined with platinum-taxane chemotherapy as first-line treatment for advanced ovarian cancer: a prospective observational study of safety and efficacy in Japanese patients (JGOG3022 trial). Int J Clin Oncol. (2019) 24:103–14. 10.1007/s10147-018-1319-y 30030657 PMC6326987

[18] Fuh KC Secord AA Bevis KS Huh W ElNaggar A Blansit K Comparison of bevacizumab alone or with chemotherapy in recurrent ovarian cancer patients. Gynecol Oncol. (2015) 139:413–8. 10.1016/j.ygyno.2015.06.041 26144600

[19] Previs RA Spinosa D Fellman BM Lorenzo A Mulder I Mahmound M Bevacizumab beyond progression: Impact of subsequent bevacizumab retreatment in patients with ovarian, fallopian tube, and peritoneal cancer after progression. J Clin Oncol. (2019) 15 suppl:5557–7. 10.1200/JCO.2019.37.15_suppl.5557 PMC12115928 39987772

[20] Lee JY Park JY Park SY Lee JW Kim JW Kim YB. Real-world effectiveness of bevacizumab based on AURELIA in platinum-resistant recurrent ovarian cancer (REBECA): A Korean Gynecologic Oncology Group study (KGOG 3041). Gynecol Oncol. (2019) 152:61–7. 10.1016/j.ygyno.2018.10.031 30409490

[21] IBM Corp Released 2010. IBM SPSS Statistics for Windows, Version 19.0. Armonk, NY: IBM Corp.

[22] Stuart GC Kitchener H Bacon M duBois A Friedlander M Ledermann J. 2010 Gynecologic Cancer InterGroup (GCIG) consensus statement on clinical trials in ovarian cancer: report from the Fourth Ovarian Cancer Consensus Conference. Int J Gynecol Cancer. (2011) 21:750–5. 10.1097/IGC.0b013e31821b2568 21543936

[23] Huang YF Kuo MT Liu YS Cheng YM Wu PY Chou CY. A dose escalation study of trientine plus carboplatin and pegylated liposomal doxorubicin in women with platinum-resistant/-refractory or partially platinum-sensitive epithelial ovarian, tubal and peritoneal cancer. Front Oncol. (2019) 9:437. 10.3389/fonc.2019.00437 31179244 PMC6544081

[24] Mirza MR Monk BJ Herrstedt J Oza AM Mahner S Redondo A. Niraparib maintenance therapy in platinum-sensitive, recurrent ovarian cancer. N Engl J Med. (2016) 375:2154–64. 10.1056/NEJMoa1611310 27717299

[25] Coleman RL Oza AM Lorusso D Aghajanian C Oaknin A Dean A. Rucaparib maintenance treatment for recurrent ovarian carcinoma after response to platinum therapy (ARIEL_3_): a randomised, double-blind, placebo-controlled, phase 3 trial [published correction appears. Lancet. (2017) 390:1949–61. 10.1016/S0140-6736(17)32440-6 28916367 PMC5901715

[26] Pujade-Lauraine E Ledermann JA Selle F Gebski V Penson RT Oza AM Olaparib tablets as maintenance therapy in patients with platinum-sensitive, relapsed ovarian cancer and a BRCA1/2 mutation (SOLO_2_/ENGOT-Ov21): a double-blind, randomised, placebo-controlled, phase 3 trial [published correction appears. Lancet Oncol. (2017) 18:1274–84. 10.1016/S1470-2045(17)30469-2 28754483

[27] Ray-Coquard I Pautier P Pignata S Pérol D González-Martín A Berger R. Olaparib plus bevacizumab as first-line maintenance in ovarian cancer. N Engl J Med. (2019) 381:2416–28. 10.1056/NEJMoa1911361 31851799

[28] Wu YH Chang TH Huang YF Chen CC Chou CY. COL11A1 confers chemoresistance on ovarian cancer cells through the activation of Akt/c/EBPβ pathway and PDK1 stabilization. Oncotarget. (2015) 6:23748–63. 10.18632/oncotarget.4250 26087191 PMC4695149

[29] Wu YH Huang YF Chang TH Chou CY. Activation of TWIST1 by COL11A1 promotes chemoresistance and inhibits apoptosis in ovarian cancer cells by modulating NF-κB-mediated IKKβ expression. Int J Cancer. (2017) 141:2305–17. 10.1002/ijc.30932 28815582

[30] Hsu KF Shen MR Huang YF Cheng YM Lin SH Chow NH. Overexpression of the RNA-binding proteins Lin28B and IGF2BP3 (IMP3) is associated with chemoresistance and poor disease outcome in ovarian cancer. Br J Cancer. (2015) 113:414–24. 10.1038/bjc.2015.254 26158423 PMC4522643

[31] Chen WC Qiu JT Lai CH Huang HJ Lin CT Chen MY. Outcomes and prognoses of patients with ovarian cancer using bevacizumab: 6-year experience in a tertiary care hospital of northern Taiwan. PLoS ONE. (2017) 12:e0175703. 10.1371/journal.pone.0175703 28467466 PMC5415172

[32] Chiang YC Chen CA Chiang CJ Hsu TH Lin MC You SL. Trends in incidence and survival outcome of epithelial ovarian cancer: 30-year national population-based registry in Taiwan. J Gynecol Oncol. (2013) 24:342–51. 10.3802/jgo.2013.24.4.342 24167670 PMC3805915

[33] Lee SP Hsu HC Tai YJ Chen YL Chiang YC Chen CA. Bevacizumab dose affects the severity of adverse events in gynecologic malignancies. Front Pharmacol. (2019) 10:426. 10.3389/fphar.2019.00426 31105567 PMC6498445

[34] Neyt M Vlayen J Devriese S Camberlin C. First- and second-line bevacizumab in ovarian cancer: A Belgian cost-utility analysis. PLoS ONE. (2018) 13:e0195134. 10.1371/journal.pone.0195134 29630612 PMC5891000

