# Supplementary material for: Real-World Study of Adding Bevacizumab to Chemotherapy for Ovarian, Tubal, and Peritoneal Cancer as Front-Line or Relapse Therapy (ROBOT): 8-Year Experience
Source: Front Oncol. 2020 Jul 14;10:1095. doi: 10.3389/fonc.2020.01095 (PMC7372289; doi:10.3389/fonc.2020.01095)
Supplement: Supplementary file 1 [file Data_Sheet_1.docx]

***Supplementary material***

The Supplementary Material for this article can be found online.

**Supplementary Figure 1.** Progression-free and overall survival curves stratified by the use of bevacizumab in subgroups of serous and clear cell histology in an early and advanced stage, tested by the log-rank test. **(A)** Early-stage clear cell carcinoma group, **(B)** early-stage serous carcinoma group, **(C)** advanced-stage clear cell carcinoma group, and **(D)** advanced-stage serous carcinoma group.

stage serous carcinoma group; **(C)** advanced-stage clear cell carcinoma group; **(D)** advanced-stage serous carcinoma group.
