# Supplementary figures and images for: Real-World Study of Adding Bevacizumab to Chemotherapy for Ovarian, Tubal, and Peritoneal Cancer as Front-Line or Relapse Therapy (ROBOT): 8-Year Experience
Source: Front Oncol. 2020 Jul 14;10:1095. doi: 10.3389/fonc.2020.01095 (PMC7372289; doi:10.3389/fonc.2020.01095)

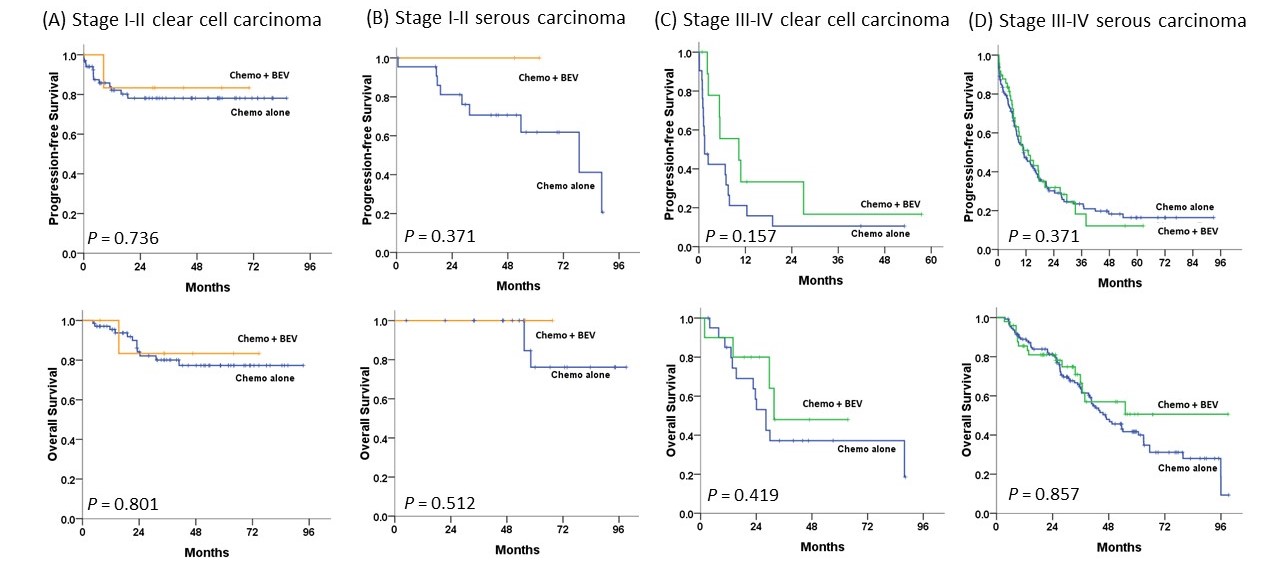

Supplement: Supplementary file 2 [file Image_1.JPEG]
